# 

**Published:** 1904-01-16

**Authors:** 


### CONTENTS.

**Doctors and Midwives** - 267

**The Regius Professorship of Medicine at Oxford** 268

**Annotations** - 269

**Notes and News** - 270

**Hospital Clinics and Medical Progress—**

What We Owe to Experiments on Animals - 271

The Dangers of Inflating the Stomach - 273

Atrophic Rhinitis - 273

Progress in Fevers - 274

Diseases of the Heart and Circulation - 275

Progress in Surgery of the Stomach - 276

**New Appliances and Things Medical** - 277

**Carol of the Maison Dieu** - 278

**The Book World of Medicine and Science** - 279

**Hospital Administration—**

Alexandra Hospital for Children, Rhyl - 280

Pensions to Asylum Officials - 281

Glances at the Hospitals - 282

Practical Departments - 283

**Editor's Letter-Box** - 284

**The Student of Medicine** - 284

*NURSING SECTION.*

**Notes on News from the Nursing World—** The Queen and the Nurses of Bakewell Infirmary—Princess Christian at the Royal Free Hospital—Sir William Bennett on Modern Nursing—The "Claims" of Army Nursing Sisters—Death of a Hongkong Matron—Croydon Guardians and the Matron's Signature—A New Year's Charge to Nurses—Chelsea Hospital for Women—Belfast Union Infirmary—A Fancy Dress Concert by Nurses—Royal County Hospital, Ryde—Hertford Hospital, Paris—An Official of the Union—A Nurse's Successful Action for Libel—Midwives' Institute—Short Items - 211-214
**The Nursing Outlook** - 215
**Sir William Bennett on Modern Nursing in Private Practice** - 216
**The State Registration of Nurses** - 218
**The Nurses' Bookshelf** - 219
**Everybody's Opinion** - 219
**Appointments** - 221
**Novelties for Nurses** - 221
**Echoes from the Outside World** - 222
**The Awakening of a Soul** - 223
**Notes and Queries** - 224
**For Reading to the Sick** - 224

